# Development and evaluation of the utility of GenoBaits Peanut 40K for a peanut MAGIC population

**DOI:** 10.1007/s11032-023-01417-w

**Published:** 2023-09-30

**Authors:** Ziqi Sun, Zheng Zheng, Feiyan Qi, Juan Wang, Mengmeng Wang, Ruifang Zhao, Hua Liu, Jing Xu, Li Qin, Wenzhao Dong, Bingyan Huang, Suoyi Han, Xinyou Zhang

**Affiliations:** Institute of Crop Molecular Breeding, Henan Academy of Agricultural Sciences/The Shennong Laboratory/State Industrial Innovation Center of Biological Breeding/Key Laboratory of Oil Crops in Huang-Huai-Hai Plains, Ministry of Agriculture/Henan Provincial Key Laboratory for Oil Crops Improvement, Zhengzhou, Henan China

**Keywords:** GenoBaits Peanut 40K, GBTS liquid chip, MAGIC population, Pod size, Peanut

## Abstract

Population and genotype data are essential for genetic mapping. The multi-parent advanced generation intercross (MAGIC) population is a permanent mapping population used for precisely mapping quantitative trait loci. Moreover, genotyping-by-target sequencing (GBTS) is a robust high-throughput genotyping technology characterized by its low cost, flexibility, and limited requirements for information management and support. In this study, an 8-way MAGIC population was constructed using eight elite founder lines. In addition, GenoBaits Peanut 40K was developed and utilized for the constructed MAGIC population. A subset (297 lines) of the MAGIC population at the S2 stage was genotyped using GenoBaits Peanut 40K. Furthermore, these lines and the eight parents were analyzed in terms of pod length, width, area, and perimeter. A total of 27 single nucleotide polymorphisms (SNPs) were revealed to be significantly associated with peanut pod size-related traits according to a genome-wide association study. The GenoBaits Peanut 40K provided herein and the constructed MAGIC population will be applicable for future research to identify the key genes responsible for important peanut traits.

**Supplementary Information:**

The online version contains supplementary material available at 10.1007/s11032-023-01417-w.

## Introduction

Peanut (*Arachis hypogaea* L.) is one of the most important oil crops worldwide (Lu et al. 2019). Developing high-quality and disease-resistant varieties with high yields has been a major goal of peanut breeding programs. Molecular marker-assisted selection (MAS) is one of the most effective plant breeding methods (Hasan et al. 2021). Elucidating the genetic basis of important peanut traits will help to improve specific characteristics of peanut cultivars through MAS. The development of next-generation sequencing technology and the availability of reference genomes for the cultivated groundnut and its ancestral species (Bertioli et al. 2016; Bertioli et al. 2019) have facilitated the mapping of genes mediating important peanut traits (Liu et al. 2020, Sun et al. 2022, Qi et al. 2022).

Single nucleotide polymorphism (SNP) arrays are robust high-throughput genotyping tools that are less expensive than next-generation sequencing platforms (Liu et al. 2022). Pandey et al. (2017) developed a high-density “Axiom_*Arachis*” genotyping array with 58K SNPs, which greatly promoted the mapping of genes related to key peanut characteristics. For example, this array was used to identify the genes mediating the resistance to late leaf spot (Moretzsohn et al. 2023). It has also been used along with a recombinant inbred line (RIL) population to reveal the genomic regions and candidate genes associated with the seed weight and shelling percentage of groundnut (Gangurde et al. 2023) as well as with the African core groundnut collection to detect novel loci for the resistance to groundnut rosette disease on the basis of a genome-wide association study (GWAS) (Achola et al. 2023). Additionally, the 58K SNP array was used to analyze the genetic diversity of Korean peanut germplasm (Nabi et al. 2021).

However, the targeted SNPs cannot be adjusted after the SNP probes are fixed in the routine chip SNP array (Liu et al. 2022). Therefore, Xu et al. (2020) developed genotyping-by-target sequencing (GBTS), which involves liquid chip technology and is characterized by its low cost, limited demands on facilities, highly flexible marker types, sharable and accumulative marker data, and limited requirements for information management and support. Moreover, this technology is widely applicable to the following areas including evaluating germplasm, constructing high-density genetic linkage maps, genetic mapping, and protecting intellectual property rights associated with crop varieties (Xu et al. 2020). To date, several GenoBaits marker panels have been developed for animals and plants, including GenoBaits Maize 20K (Guo et al. 2019), GenoBaits Rice 10K (Hussain et al. 2022), GenoBaits Soy40K (Liu et al. 2022), GenoBaits Wheat 16K (Huang et al. 2022), and GenoBaits Porcine SNP 50K (Wang et al. 2022).

Conventionally, populations used for quantitative trait locus (QTL) mapping have included RIL populations, doubled haploid backcrossed populations, or F_2_ populations derived from two parents, which can only be used to analyze two alleles and limits the genetic recombination and resolution for detecting QTLs (Bandillo et al. 2013). To overcome the limitations of bi-parental populations, a multi-parent advanced generation intercross (MAGIC) strategy was initially proposed for crops by Mackay and Powell (2007). This strategy can be used to analyze multiple alleles and to increase recombination rates and mapping resolutions (Cavanagh et al. 2008). Several MAGIC populations are available for diverse crops, including rice (Meng et al. 2016), maize (Dell'Acqua et al. 2015), and wheat (Stadlmeier et al. 2018). These populations have been used for the high-resolution dissection of the QTLs and genes responsible for complex agronomic traits.

To identify the genes associated with important peanut traits and develop useful markers for breeding, a MAGIC population was constructed and GenoBaits Peanut 40K was developed in this study. The objectives of this study were to (1) construct an 8-way MAGIC population using eight elite founder lines; (2) develop a liquid chip array GenoBaits Peanut 40K for peanut; (3) conduct a genetic analysis for the eight founder lines and the MAGIC population at the S2 stage; and (4) perform a GWAS for four traits related to peanut pod size using the MAGIC population at the S2 stage and its parents.

## Materials and methods

### Eight founder lines used for developing the MAGIC population

Eight elite peanut varieties or germplasms, namely, Yuanza9102 (N730), Zhonghua6 (N709), Yuhua15 (N734), Weihua8 (N743), Yueyou20 (N745), Fuhuasheng (N744), Silihong (N741), and NC94022 (N739) (Table 1), which were designated as A, B, C, D, E, F, G, and H, respectively, were used as the founder lines to develop the MAGIC population. These eight founder lines represent three of the five botanical varieties of peanut (i.e., ssp. *fastigiata* var. *vulgaris*, ssp. *fastigiata* var. *fastigiata*, and ssp. *hypogaea* var. *hypogaea*) and two kinds of irregular peanut types (i.e., irregular *fastigiata*-type and irregular *hypogaea*-type) (Table 1). Five of these lines (A–E), which were widely grown varieties developed by breeders in four major peanut-producing provinces in China, have high yield potentials and oil contents, are resistant to diseases, and exhibit other specific characteristics (e.g., wide adaptability, high shelling percentage, and deep pod mesh) (Table 1). Fuhuasheng and Silihong (F, G) are landraces originated from Shandong and Liaoning provinces in China, respectively (Table 1). Fuhuasheng is one of the most prominent parental varieties included in the pedigrees of most peanut varieties in China. Silihong, which produces pods that typically contain three or four seeds with red seed coat, is widely cultivated in northeastern China. NC94022 (H) is a late-maturing breeding line with a prostrate growth habit and originated in the USA (Shrestha et al. 2013).

**Table 1:** Characteristics of the eight founder lines used for developing the MAGIC population

ID	Germplasm/variety	Variant	Type	Origin	Characteristics
N730	Yuanza9102 (A)	Irregular *fastigiata*-type	Breeding line	Henan, China	A variety, derived from an interspecific cross combination, with high oil content, bacterial wilt resistance, and wide adaption
N709	Zhonghua6 (B)	subsp. *fastigiata* var. *vulgaris*	Breeding line	Hubei, China	Early maturity variety with small seed size, bacterial wilt resistance, and pale green leaves
N734	Yuhua15 (C)	Irregular *hypogaea*-type	Breeding line	Henan, China	High yielding variety with good combining ability and high oil content, progenitor of many breeding lines
N743	Weihua8 (D)	Irregular *hypogaea*-type	Breeding line	Shandong, China	High yielding variety with moderate pod size and thin pod shell
N745	Yueyou20 (E)	subsp*. fastigiata* var. *vulgaris*	Breeding line	Guangdong, China	High resistance to leaves diseases, thick shell, and deep pod mesh on peanut shell
N744	Fuhuasheng (F)	Irregular *hypogaea*-*type*	Landrace	Shandong, China	One of the main progenitors of Chinese peanut varieties, in the pedigrees of most varieties of China with deep pod waist
N741	Silihong (G)	subsp. *fastigiata* var. *fastigiata*	Landrace	Liaoning, China	Multi-seeds in one pod with red seed coat and low number of branches
N739	NC94022 (H)	subsp. *hypogaea* var. *hypogaea*	Breeding line	America	Late maturity with prostrate growth habit, small size seed, and light pod mesh

### SNP selection and array design for GenoBaits Peanut 40K

A diverse set comprising 353 peanut germplasms that underwent a whole-genome re-sequencing (20×) analysis was used to select SNPs. Approximately 0.93 million high-quality SNPs and insertions/deletions (*Arachis hypogaea* cv. Tifrunner version 1) were identified after the quality control and filtering: missing rate > 0.05 (any alleles with fewer than five supporting reads were marked as missing), minor allele frequency (MAF) < 0.01, and number of heterozygous alleles > 10 (Zheng et al. 2022). The SNP sites were selected according to the following criteria: (1) unique for each of the eight founder lines used as the parents of the MAGIC population (e.g., the genotype of one parent was A:A, whereas the genotype of the other seven parents was G:G); (2) evenly distributed across 20 chromosomes (as much as possible). The selected SNP sites were evaluated by MolBreeding Biotechnology Co., Ltd. (Shijiazhuang, China). Probes that were designed on the basis of the flanking sequences and targeted capture sequencing technology were subsequently synthesized*.* The effects of the selected SNPs on genes were predicted using SNPEff v5.0 (Cingolani et al. 2012).

### Plant materials and phenotypes

The 297 S2 plants derived from one 8-way cross (A/E//D/G///B/C//F/H) and further two generation of single seed descent and the eight founder lines were used to evaluate GenoBaits Peanut 40K and the utility of the MAGIC population. The seeds of 297 S2 plants and eight founder lines were harvested and pod size-related characteristics (i.e., area, perimeter, length, and width) were measured using the SC-G software (Hangzhou Wanshen Detection Technology Co., Ltd., China). Because of the low reproduction efficiency of peanut, an average of approximately six pods was used for the phenotypic evaluation of each plant.

### DNA isolation and genotyping with GenoBaits Peanut 40K

Genomic DNA was extracted from young unfolded leaves using the Plant Genomic DNA Extraction Kit (Tiangen Biotech, Beijing, China). The purity and integrity of the extracted DNA was evaluated by 1% agarose gel electrophoresis, whereas the DNA concentration was precisely determined using Qubit. The high-quality DNA samples were sequenced using GenoBaits Peanut 40K by MolBreeding Biotechnology Co., Ltd. (Shijiazhuang, China). The raw data were filtered for quality using the FASTQ software (Chen et al. 2018) and then aligned to the peanut reference genome (*Arachis hypogaea* cv. Tifrunner version 1) using the BWA software (Li and Durbin 2009). The standard pipeline of the GATK software (Poplin et al. 2018) was used to detect SNPs for genotyping. Finally, the SNP set was filtered according to the following parameters: missing rate < 0.3 and MAF > 0.05.

### Diversity and population structure analyses and GWAS

The diversity of the 297 MAGIC lines and eight founder lines was analyzed using the UPGMA algorithm implemented in the TASSEL v5.0 software (Bradbury et al. 2007). The phylogenetic tree was drawn using the online program iTOL v6.7.3 (Letunic and Bork 2021). The population structure was deduced using ADMIXTURE v1.30 (*K* = 1–20) (Alexander and Lange 2011). The mixed linear model (MLM) implemented in TASSEL v5.0 (Bradbury et al. 2007) was used for the association analysis and the GWAS threshold was set as 0.05/*n*, with *n* representing the number of markers.

## Results

### Construction of a MAGIC population for peanut

A population was obtained from an 8-way cross involving the eight elite founder lines (Table 1). According to the method described by Bandillo et al. (2013), a half-allele mating system was used for the three stages required for the construction of the MAGIC population. At the first stage, 28 bi-parental crosses were conducted by inter-mating the eight founder lines. To obtain enough hybrids for the subsequent crosses, 30 seeds from each parent were sown. The resulting 28 F_1_ lines were inter-crossed for the 4-way cross (i.e., all 210 of the possible crosses). The combinations were set so that no parent was represented more than once in the 4-way cross. The 210 4-way F_1_ lines were inter-crossed for the 8-way cross (i.e., all 315 possible crosses were completed in the same manner).

For the 8-way cross, 4–36 confirmed hybrids were obtained from each of the crosses and advanced by selfing, with an average of approximately 250 seeds harvested per cross at the S2 stage. A subset of the 8-way cross consisting of 35 crosses with a population size of approximately 200 (or 500 for one cross) was selected and used for advancing generations via single seed descent. Thus, the target population comprised 7000 lines (i.e., 35 × 200). The subset was selected in such a manner that only one of the nine possible crosses was chosen (e.g., one of A/B//C/D///E/F//G/H, A/B//C/D///E/G//F/H, A/B//C/D///E/H//F/G, A/C//B/D///E/F//G/H, A/C//B/D///E/G//F/H, A/C//B/D///E/H//F/G, A/D//B/C///E/F//G/H, A/D//B/C///E/G//F/H, and A/D//B/C///E/H//F/G). The other crosses were stored at −20 °C for later use.

### SNP selection and array design

**Fig. 1:**
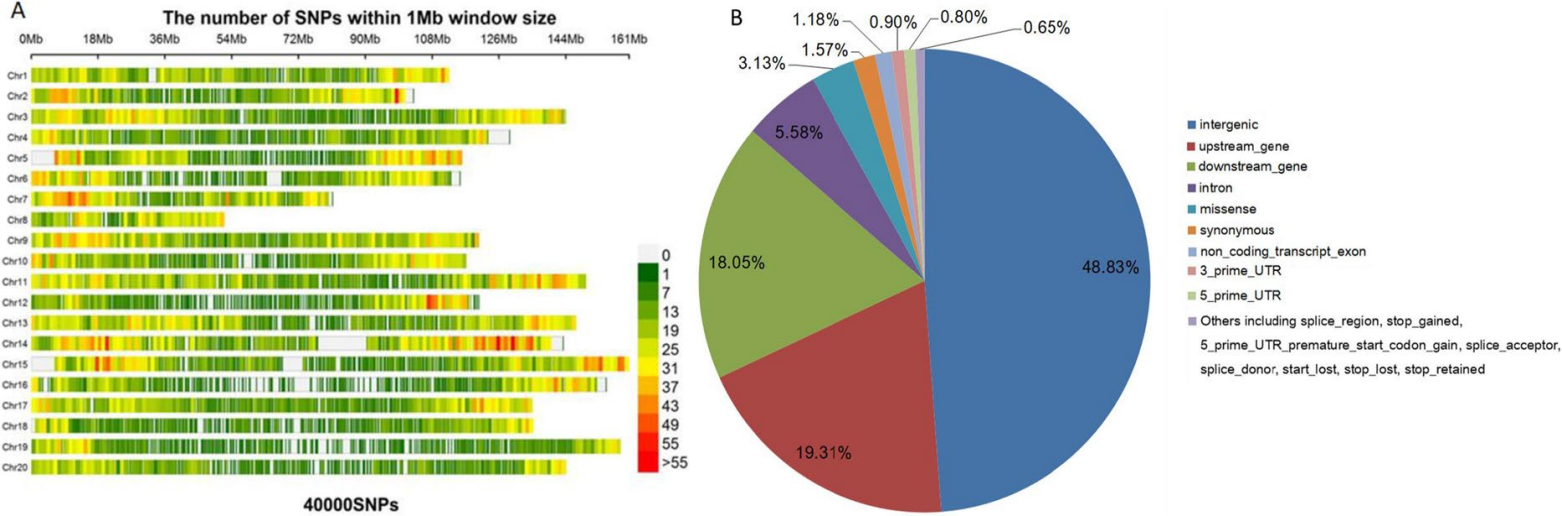
Distribution of the 40K SNPs on 20 chromosomes (**A**) and genomic positions of selected SNPs (**B**)

To genotype the MAGIC population, GenoBaits Peanut 40K was developed using liquid chip technology. The 40,000 SNPs that were selected from the variation set of 353 peanut germplasms (Supplementary Table S1) were evenly distributed across the 20 peanut chromosomes (Fig. 1A). The number of SNPs per chromosome ranged from 1070 (Arahy.08) to 3029 (Arahy.14), with an average of one SNP per 63,457 bases (Table 2). In terms of their genomic positions, 48.83% of the SNPs were located in intergenic regions, but the SNPs were also present in the following locations: upstream_gene (19.31%), downstream_gene (18.05%), intron (5.58%), missense (3.13%), synonymous (1.57%), non_coding_transcript_extron (1.18%), 3_prime_UTR (0.90%), 5_prime_UTR (0.80%), and others (0.65%) including splice_region, stop_gained, 5_prime_UTR_premature_start_codon_gain, splice_accepter, splice_donor, start_lost, stop_lost, and stop_retained (Fig. 1B).

**Table 2:** Number of variants on 20 chromosomes

Chromosome	Length	Variants	Variants rate
arahy.Tifrunner.gnm1.Arahy.01	112,420,854	2009	55,958
arahy.Tifrunner.gnm1.Arahy.02	102,981,163	1804	57,084
arahy.Tifrunner.gnm1.Arahy.03	143,813,506	2570	55,958
arahy.Tifrunner.gnm1.Arahy.04	128,801,742	1730	74,451
arahy.Tifrunner.gnm1.Arahy.05	115,930,344	1933	59,974
arahy.Tifrunner.gnm1.Arahy.06	115,504,342	1779	64,926
arahy.Tifrunner.gnm1.Arahy.07	81,119,488	1763	46,012
arahy.Tifrunner.gnm1.Arahy.08	51,897,010	1070	48,501
arahy.Tifrunner.gnm1.Arahy.09	120,519,698	2463	48,932
arahy.Tifrunner.gnm1.Arahy.10	117,088,237	1828	64,052
arahy.Tifrunner.gnm1.Arahy.11	149,299,306	2788	53,550
arahy.Tifrunner.gnm1.Arahy.12	120,579,088	1534	78,604
arahy.Tifrunner.gnm1.Arahy.13	146,725,006	2614	56,130
arahy.Tifrunner.gnm1.Arahy.14	143,237,272	3029	47,288
arahy.Tifrunner.gnm1.Arahy.15	160,879,708	2543	63,263
arahy.Tifrunner.gnm1.Arahy.16	154,808,347	1662	93,145
arahy.Tifrunner.gnm1.Arahy.17	134,922,436	2249	59,992
arahy.Tifrunner.gnm1.Arahy.18	135,150,084	1453	93,014
arahy.Tifrunner.gnm1.Arahy.19	158,625,764	1441	110,080
arahy.Tifrunner.gnm1.Arahy.20	143,980,330	1738	82,842
Total	2,538,283,725	40,000	63,457

To more precisely genotype the MAGIC population, 30,082 sites that were specific to one of the eight founder lines were designated as 1:7 (i.e., the genotype of one parent differed from that of the other seven parents), whereas 9918 sites were designated as 2:6 (i.e., the genotype of two parents differed from that of the other six parents) to ensure the 40,000 SNPs were evenly distributed on the 20 chromosomes (Supplementary Table S2). The founder line with the most unique sites was N741, followed by N745. The founder lines with the fewest unique sites were N734 and N743 (Supplementary Table S2). The number of polymorphic SNPs between each pair of the eight founder lines (28 combinations) ranged from 1815 (between N739 and N744) to 18,458 (between N741 and N745) (Supplementary Table S3). Probes were designed for each SNP in both the forward and reverse direction, but three primers were designed for nine SNP sites (Supplementary Table S4).

### Accuracy of the GBTS technology

The accuracy of the GBTS technology was evaluated by comparing the genotypes of the eight founder lines revealed by GenoBaits Peanut 40K and the previously reported genotypes determined on the basis of whole-genome resequencing technology (Zheng et al. 2022). More specifically, the number of consistent SNPs between the two technologies was divided by the total number of SNP (i.e., 40,000). The accuracy ranged from 96.57 to 99.33% for N709, N730, N734, N743, N744, and N745, while those of the other two founder lines (N739 and N741) was only about 84% (Table 3). The lower accuracy for N739 and N741 was likely due to excessive heterozygous and missing sites, respectively (Table 3), which may be related to the differences between the genomes of these two lines and the reference genome.

**Table 3:** Comparison of the genotypes of eight founder lines determined using liquid chip technology and sequencing data

Variety	N709	N730	N734	N739	N741	N743	N744	N745
The number of SNPs different with sequencing								
Homozygous	23	11	19	663	49	23	18	28
Heterozygous	61	39	42	4825	199	34	39	70
Indel	3	2	2	0	2	1	1	1
Missing	586	574	205	1232	6076	217	1315	582
The number of SNPs consistent with sequencing	39,327	39,374	39,732	33,280	33,674	39,725	38,627	39,319
Accuracy	98.32%	98.44%	99.33%	83.20%	84.19%	99.31%	96.57%	98.30%

### Genetic analysis of the MAGIC population at the S2 stage

**Fig. 2:**
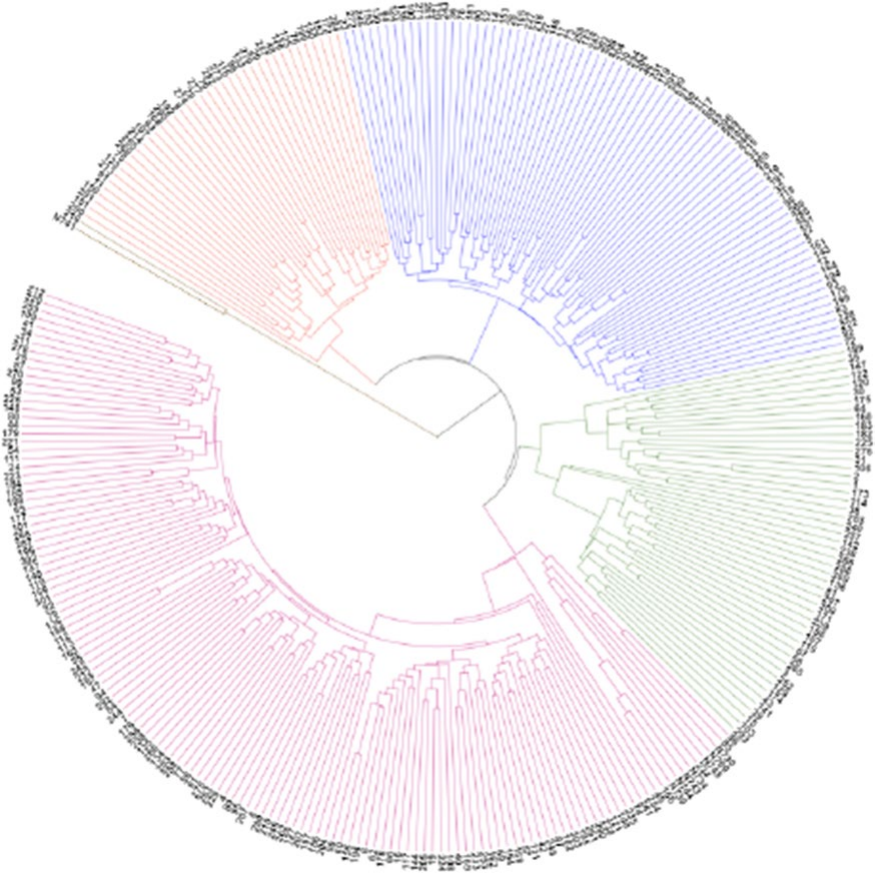
Phylogenetic tree comprising the 297 lines of the MAGIC population at the S2 stage and eight founder lines

The 297 lines of the 8-way cross were genotyped at the S2 stage using GenoBaits Peanut 40K. A total of 18,816 filtered SNPs with a missing rate < 0.3 and MAF > 0.05 were used for the genetic analysis. A phylogenetic tree with the 297 lines and eight founder lines was constructed. The 305 lines were roughly divided into five clusters, which were differentiated by color in the phylogenetic tree (brown, red, blue, green, and purple) (Fig. 2). The parent N741 and line 216 were clustered into clade 1 and were far away from the other seven parents (clade 5) (Fig. 2), which due to that N741 is a landrace from ssp. *fastigiata* var. *fastigiata* and exhibits a huge genetic difference with other seven parents (i.e., 13,655 unique sites in Supplementary Table S2). Among the seven parents in clade 5, three parents from ssp. *fastigiata* (N709, N730, and N745) were grouped together and then clustered with the parents from ssp. *hypogaea* (N734, N743, N744, and N739) (Fig. 2). Except 216, the eight founder lines were not cluster together with the S2 lines, the reason may be that the S2 population is still highly heterozygous as well as the parents are homozygous.

**Fig. 3:**
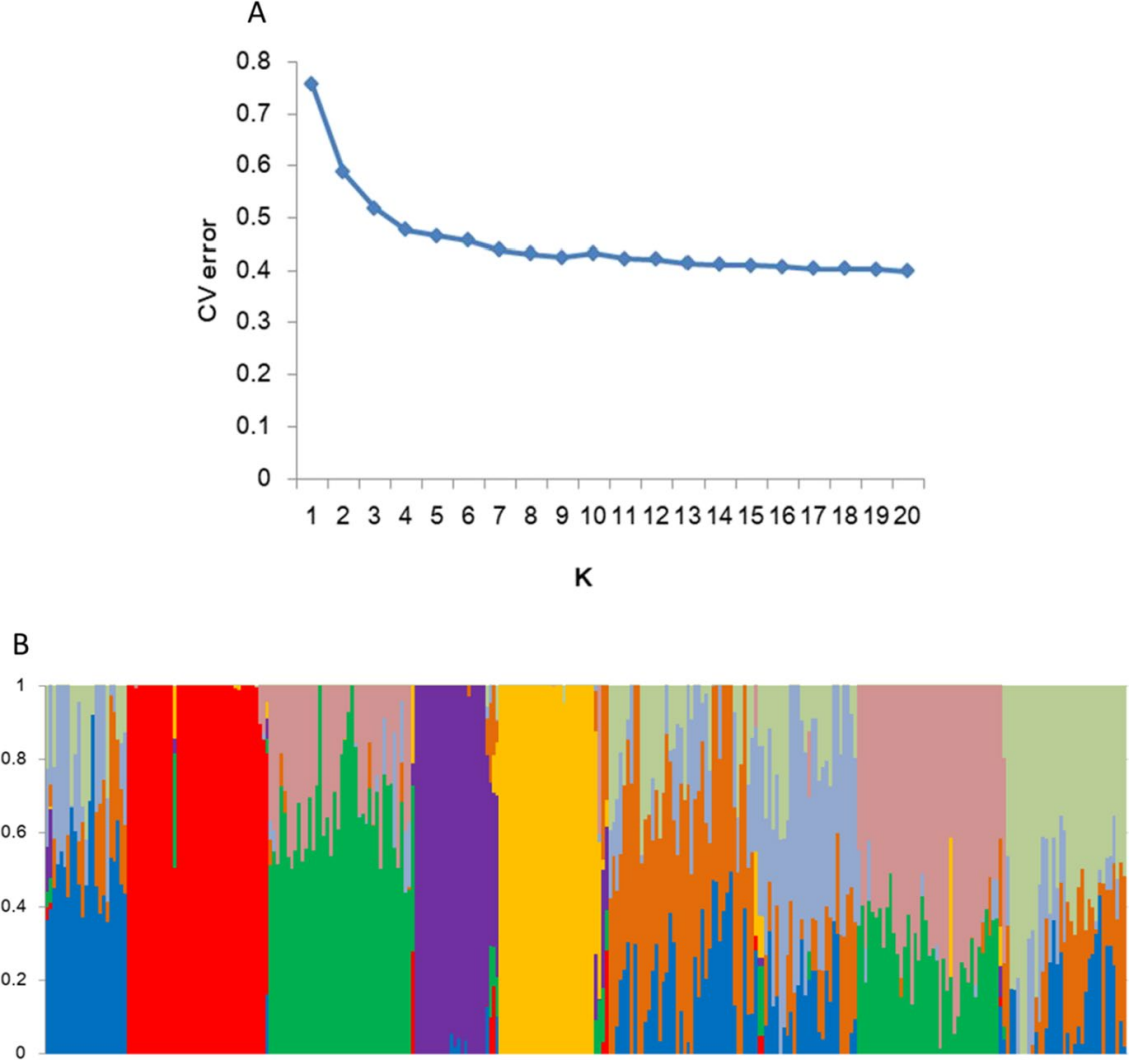
CV error for *K* = 1–20 (**A**) and population structure for *K* = 9 (**B**)

Population structures were analyzed using ADMIXTURE v1.30, with *K* = 1–20. The 305 lines were grouped into nine clusters because the CV error reached the smallest when *K* = 9 (Fig. 3A–B). The eight founder lines were grouped into five clusters, with N709, N730, and N741 in separate clusters, N734, N739, and N745 in the same cluster, and N743 and N744 in another cluster.

### Genome-wide association study for pod size-related traits

**Fig. 4:**
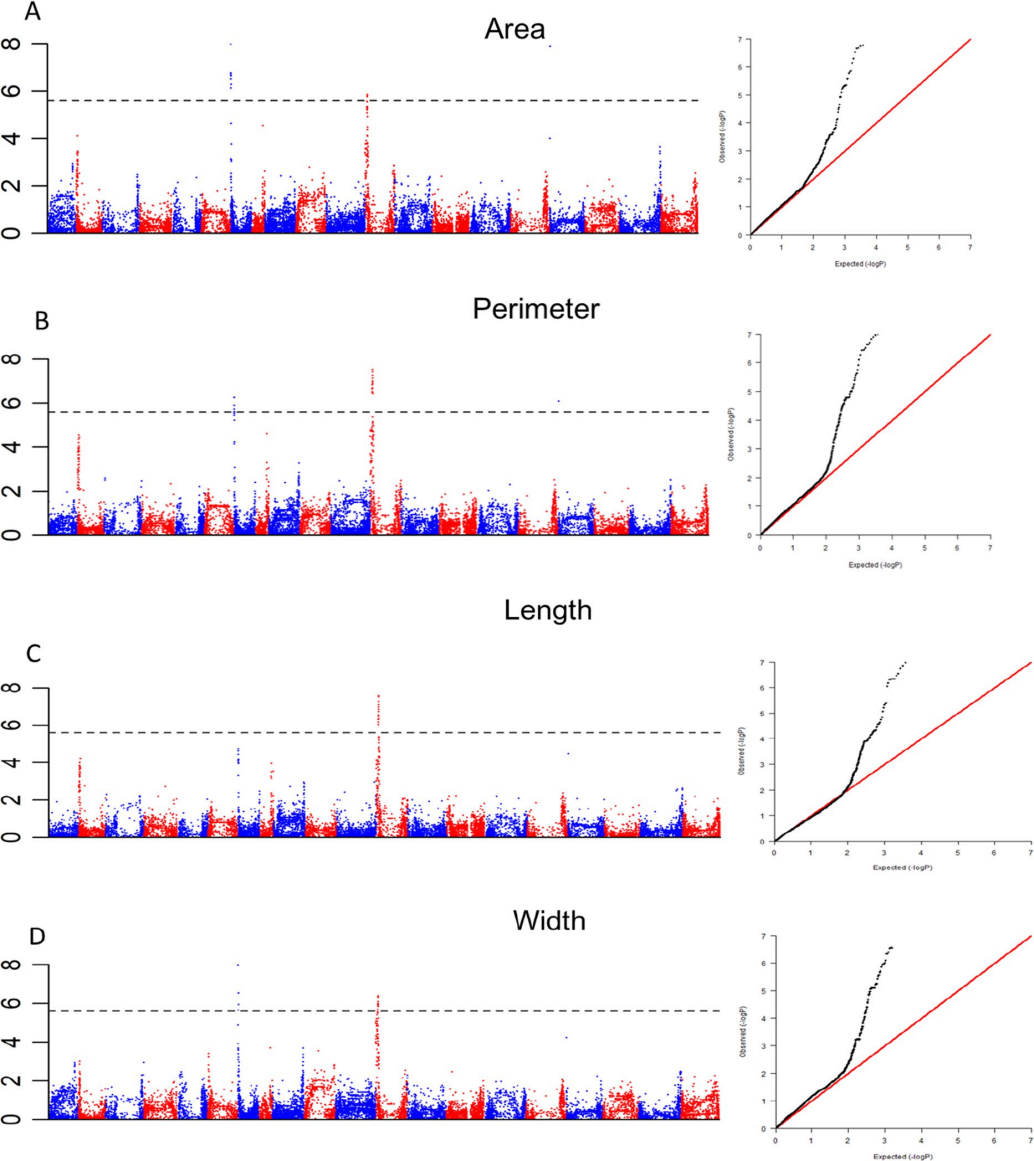
Manhattan and QQ plots for peanut pod area (**A**), perimeter (**B**), length (**C**), and width (**D**)

The 18,816 filtered SNPs were screened for SNPs significantly associated with the peanut pod area, perimeter, length, and width according to the MLM model. The Q file for *K* = 9 generated during the population structure analysis was used as the covariate (Q) in the MLM model. Kinship (*K*) was calculated using TASSEL v5.0. A total of 27 SNPs significantly associated with at least two of the four pod size-related traits were identified at the threshold of 5.50 [−log(0.05/18,186)] (Table 4, Fig. 4, and Supplementary Table S5). Of these SNPs, 10 were on chromosome 7, 16 were on chromosome 12, and one was on chromosome 17 (Table 4 and Supplementary Table S5).

**Table 4:** Results for the GWAS of peanut pod size-related traits

Chromosome	Position	−log(*P*)	add_effect	Trait
7	130688	6.27–10.61	−	Area, perimeter, width
7	292285	6.27–10.70	+	Area, perimeter, width
7	492470	5.72–7.97	+	Area, width
7	586747	5.91–10.80	+	Area, perimeter, width
7	811108	5.63–9.89	+	Area, perimeter, width
7	848538	6.75–9.76	−	Area, width
7	890383	5.55–8.87	−	Area, perimeter, width
7	920314	5.74–9.24	−	Area, perimeter, width
7	1008387	6.30–8.40	+	Area, width
7	1056718	5.61–9.12	+	Area, perimeter, width
12	7302293	5.57–6.65	+	Perimeter, length, width
12	7423728	5.57–6.65	−	Perimeter, length, width
12	7926457	5.51–6.53	−	Perimeter, length, width
12	7964822	5.70–6.48	−	Perimeter, length, width
12	8555800	5.58–6.83	+	Area, perimeter, length, width
12	8695703	5.83–7.26	−	Area, perimeter, length, width
12	8746767	6.01–6.89	+	Perimeter, length, width
12	8817149	5.95–6.70	−	Perimeter, length, width
12	8913786	5.88–6.97	+	Perimeter, length, width
12	9170907	5.86–7.09	−	Perimeter, length, width
12	9387102	5.57–7.28	−	Area, perimeter, length, width
12	9486492	5.78–7.58	+	Area, perimeter, length, width
12	9610185	5.86–7.53	−	Area, perimeter, length, width
12	9723588	5.86–6.45	−	Perimeter, width
12	9837499	5.86–6.45	−	Perimeter, width
12	9911046	5.86–6.45	−	Perimeter, width
17	625720	6.10–10.61	+	Area, perimeter, width

**Fig. 5:**
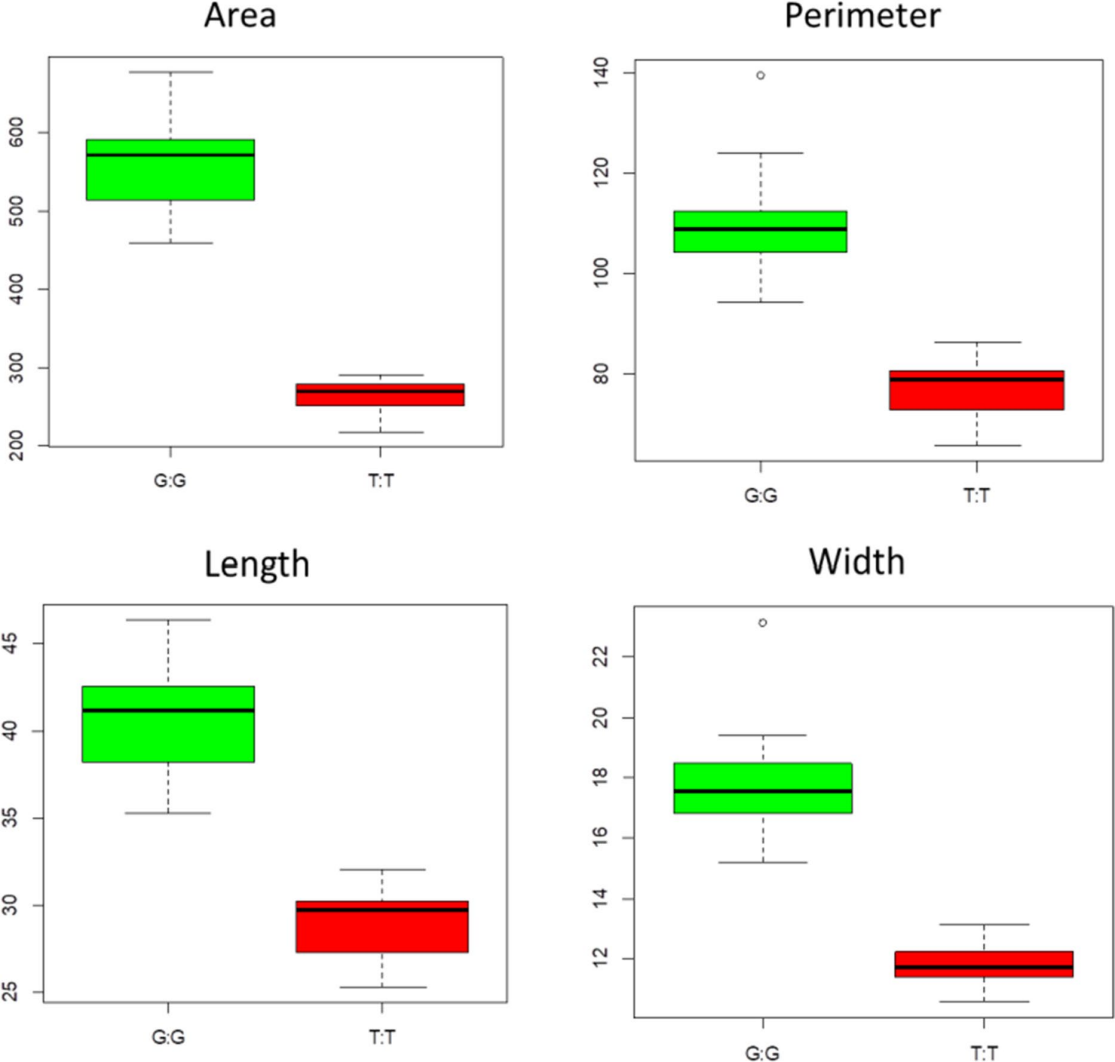
Differences in the peanut pod area, perimeter, length, and width between lines with different genotypes (G:G or T:T) at Arahy07:292285, but the same genotype (C:C) at Arahy12:9486492

The significant SNPs on chromosomes 7 and 12 were linked, respectively. The site Arahy17:625720 was linked with the identified SNPs on chromosome 7. There were significant phenotypic differences between the lines with the genotype of G:G and T:T at the site Arahy07:292285. Most lines had the same genotype at the significant sites on chromosome 12. For example, C:C was the common genotype at Arahy12:9486492, but 14 lines with small pods had a different genotype at this site (i.e., T:T). Notably, among the lines with C:C at Arahy12:9486492, the lines with G:G had significantly larger pods than those with T:T at Arahy07:292285 (Fig. 5). The genotypes of N734 and N739 differed from those of the other six parents at the significant SNP sites on chromosomes 7 and 17. Additionally, the genotype of N741 differed from those of the other seven parents at the significant SNP sites on chromosome 12.

### Candidate genes for pod size

The significant SNPs on chromosome 7 covered a physical region comprising 0.93 Mb (Table 4 and Supplementary Table S5) that contained 31 candidate genes, including those encoding a fasciclin-like arabinogalactan family protein (*Arahy.P7DY53*), a transcriptional regulator (*STERILE APETALA-like*; *Arahy.5EZV1I*), and a transcription factor subunit (*NFYB/HAP3*) (Supplementary Table S6). A total of 47 candidate genes were identified in the region (2.61 Mb) covered by the significant SNPs on chromosome 12. Some of the key genes in this region encoded a C6HC-type zinc finger RING/U-box protein (*Arahy.UZLY68*), a homeobox-leucine zipper protein (*Arahy.V0IP08*), and a MYB transcription factor (*Arahy.7ML2J7*) (Supplementary Table S6). The significant SNP on chromosome 17 was located in the exon of *Arahy.5QP4QH*, which encodes a rho GDP-dissociation inhibitor 1-like protein (Supplementary Table S6).

## Discussion

### Applicability of GenoBaits Peanut 40K

The greatest advantage of liquid chip technology-based marker panels over the alternatives is that the number of markers (e.g., 10, 20, and 40K) can vary depending on how the marker panels are being used. Moreover, they are useful for genotyping regardless of the number of samples (i.e., unlimited sample size) (Xu et al. 2020). Although GenoBaits Peanut 40K was designed for the MAGIC population, it has many other uses. For example, it is applicable for evaluating germplasm diversity, performing a linkage analysis of bi-parental populations, and conducting GWAS. The number of polymorphic SNPs between almost each pair of the two parents exceeded 3000 (approximately 9900 on average), ensuring to some extent its wide applicability (Supplementary Table S3).

The GenoBaits Peanut 40K panel may be used to analyze most peanut germplasms, although there are a few exceptions. The accuracy of the genotyping of N741 and N739 was relatively low (i.e., 84%) because of the number of missing or heterozygous sites in N741 (6076 missing sites) and N739 (4825 missing sites and 1232 heterozygous sites) (Table 3). This may be associated with the fact that N741 belonged to ssp. *fastigiata* var. *fastigiata* and one parent of N739 belonged to ssp. *hypogaea* var. *hirsuta* (Shrestha et al. 2013), the genome of which may differ substantially from the reference genome used in the present study. Therefore, an increase in sequencing depth may be required to capture the missing sites.

### Benefits of the MAGIC population

Constructing MAGIC populations is a new approach for exploiting the diversity in plant genetic resources (Arrones et al. 2020). These populations are very useful for dissecting complex traits, selecting elite lines for breeding, and constructing genomic prediction models (Arrones et al. 2020; Puglisi et al. 2021). The identified region on chromosome 7 was consistent with the QTL reported by Alyr et al. (2020). The candidate gene identified on chromosome 17 was mapped to the significant region of chromosome 7 using the updated reference genome. To the best of our knowledge, the identified region on chromosome 12 has not been reported. The significant SNPs on chromosome 7 may resulted from the difference between two parents (N734 and N739) and the other six parents, whereas the sites identified on chromosome 12 may due to the difference between N741 and the other seven parents. In addition to pod size, the constructed MAGIC population may also be used to investigate other important peanut traits because the progenies of the population vary in terms of growth habit, seed coat color, pod shell type, oil content, and other characteristics.

### Functions of the candidate genes

A total of 79 candidate genes influencing pod size were detected in the three significant regions (Supplementary Table S6). The fasciclin-like arabinogalactan family protein gene (*Arahy.P7DY53*) has been reportedly related to fundamental aspects of embryogenesis and seed development across angiosperms (Costa et al. 2019). A previous study showed that this gene is involved in the regulation of the *Brassica napus* L. silique length (Wang et al. 2019). The transcriptional regulator *STERILE APETALA-like* and the F-box domain encoded by *Arahy.5EZV1I* was reported to regulate the peanut pod and seed sizes (Alyr et al. 2020). Furthermore, *Arahy.UZLY68* encodes a C6HC-type zinc finger RING/U-box protein that may modulate the peanut pod size via ubiquitination according to a recent report on rice (Yang et al. 2021). The homeobox-leucine zipper protein gene (*Arahy.V0IP08*) affects maize kernel size and weight (Sun et al. 2022). The MYB transcription factor gene (*Arahy.7ML2J7*) controls the size of *Arabidopsis thaliana* seeds (Zhang et al. 2013). Additionally, the possibility the gene encoding a rho GDP-dissociation inhibitor 1-like protein (*Arahy.5QP4QH*) may affect peanut pod size is supported by the findings of an earlier study, which revealed the Rho-family GTPase-encoding gene *OsRac1* controls rice grain size and yield by regulating cell division (Zhang et al. 2019).

### Supplementary information

ESM 1 (XLSX 9234 kb)

## Data Availability

All data generated or analyzed during this study are included in this published article and its supplementary information files. The clean resequencing data obtained in this study are available at the BioProject database at China National Center for Bioinformation under the BioProject ID: PRJCA019839 (https://ngdc.cncb.ac.cn/gsub/submit/bioproject/PRJCA019839). Materials used in this study are available from the corresponding authors.
